# Multilabel Acoustic Event Classification Using Real-World Urban Data and Physical Redundancy of Sensors

**DOI:** 10.3390/s21227470

**Published:** 2021-11-10

**Authors:** Ester Vidaña-Vila, Joan Navarro, Dan Stowell, Rosa Ma Alsina-Pagès

**Affiliations:** 1 GTM—Grup de Recerca en Tecnologies Mèdia, La Salle Ramon Llull Univeristy, 08022 Barcelona, Spain; rosamaria.alsina@salle.url.edu; 2 GRITS—Grup de Recerca en Internet Techologies and Storage, La Salle Ramon Llull Univeristy, 08022 Barcelona, Spain; jnavarro@salleurl.edu; 3 Department of Cognitive Sciences & Artificial Intelligence, Tilburg University, 5037 AB Tilburg, The Netherlands; d.stowell@tilburguniversity.edu

**Keywords:** acoustic event classification, urban sound monitoring, multilabel classification, deep neural networks, physical redundancy, distributed computing

## Abstract

Many people living in urban environments nowadays are overexposed to noise, which results in adverse effects on their health. Thus, urban sound monitoring has emerged as a powerful tool that might enable public administrations to automatically identify and quantify noise pollution. Therefore, identifying multiple and simultaneous acoustic sources in these environments in a reliable and cost-effective way has emerged as a hot research topic. The purpose of this paper is to propose a two-stage classifier able to identify, in real time, a set of up to 21 urban acoustic events that may occur simultaneously (i.e., multilabel), taking advantage of physical redundancy in acoustic sensors from a wireless acoustic sensors network. The first stage of the proposed system consists of a multilabel deep neural network that makes a classification for each 4-s window. The second stage intelligently aggregates the classification results from the first stage of four neighboring nodes to determine the final classification result. Conducted experiments with real-world data and up to three different computing devices show that the system is able to provide classification results in less than 1 s and that it has good performance when classifying the most common events from the dataset. The results of this research may help civic organisations to obtain actionable noise monitoring information from automatic systems.

## 1. Introduction

Acoustic noise (or noise pollution) can be defined as any sound that is loud or unpleasant enough that causes some kind of disturbance [1]. Noise pollution is one of the major concerns for the European population, especially for citizens living in urban environments [2], which is materialized in an ever-rising number of complaints to public administrations [3]. This issue is further stressed on those residential areas located close to aggressive noise pollutants such as airports, railways, or highways [4]. In fact, according to the World Health Organization (WHO) [2,4], there is a worrying portion of the European population that is systematically exposed to harmful levels of noise pollution. Concretely, it is estimated that from all the citizens living in the European Union (EU), about 40% are exposed to road traffic noise levels above 55 dB(A), about 20% are exposed to levels above 65 dB(A) in daytime, and over 30% are exposed to noise levels exceeding 55 dB(A) at nighttime [5].

Continuous exposure to environmental noise pollution may result in adverse effects on health, ranging from moderate disturbances such as difficulties in understanding a voice message to chronic illnesses such as cardiovascular diseases (e.g., myocardial infarction), cognitive impairment in children, psychological disorders derived from lack of rest or sleep, or tinnitus [4,6]. In fact, according to the European Environment Agency (EEA), it is estimated that, only in Europe, 12,000 premature deaths are associated with long-term noise exposure each year. Moreover, in their latest report [7], it is estimated that more than 28 million people suffer from the aforementioned health effects derived from overexposure to noise.

In order to overcome this situation, a set of recommendations have been established (e.g., Environmental Noise Directive 2002/49/EC [8] from the European Commission or the Environmental Noise Guidelines for the European Region from the WHO [9]) to define the thresholds on the maximum amount of noise that should be perceived by citizens. For instance, the WHO distinguishes up to five different types of noise sources (i.e., road traffic noise, railway noise, aircraft noise, wind turbine noise, and leisure noise) and recommends different noise thresholds for each source depending on the time of the day (i.e., day, night) [10]. From these recommendations, it can be inferred that not all sound sources have the same impact on human disturbance. In fact, the sound level is not the only parameter that indicates the extent and intensity of noise pollution [11]. Therefore, identifying the sources of those potentially harmful sounds has emerged as a hot research topic nowadays.

So far, several efforts have been made by private and public entities on identifying acoustically polluted environments in urban areas [12]. Typically, this is done by either analyzing the distribution of noise-related complaints in a certain area or by deploying a Wireless Acoustic Sensor Network (WASN) to automatically monitor the soundscape [12]. Both approaches entail the same main underlying challenges:

1. Identifying multiple concurrent noise sources that populate a given soundscape. Typically, in real-world environments, several sounds occur simultaneously. This complicates the task of building a reliable automatic sound classifier system specialized in identifying a predefined set of acoustic events [13].
2. Monitoring large-scale urban areas in a cost-effective way. Populating (with either automatic devices or human resources) extensive urban environments requires a considerable amount of resources. For instance, it has been reported [12] that the Department of Environmental Protection from New York City employs about 50 highly qualified sound inspectors. In addition, the starting price of autonomous nodes to continuously monitoring sound is usually around EUR 1000 [14].
3. Real-time processing. Although continuous exposure to noise is harmful, short-term exposure to sporadic noise shall not be neglected. In fact, sometimes noise violations are sporadic (i.e., they last a few minutes or hours at most). Therefore, human-based noise complaint assessment systems result in being ineffective due to the fact that technicians may arrive way after the disturbance has finished [12]. Furthermore, the large amount of data to be processed by autonomous acoustic sensors may make this kind of approach challenging.

The purpose of this paper is to present an automatic classification system for acoustic events in urban environments able to address the aforementioned challenges. The proposed approach combines and improves (1) the advances of our previous work in the conception of a WASN architecture for single-label classification using physical redundancy of low-cost sensors and synthetically generated audio files [15], and (2) the outlined automatic multilabel classification system for acoustic events that the authors presented in [16]. The resulting system presented in this work features a two-stage classifier that analyzes real-world acoustic frames in real time to distinguish all the events that appear in them—not only on the foreground soundscape but also on the background. It is understood that events in the foreground are those with more saliency than the average noise. Similarly, events in the background are those events with similar saliency to the average noise.

The first stage is composed of a deep neural network that has been trained to identify different events that may occur concurrently (also referred to as a multilabel classification). The second layer is aimed at aggregating the first-stage classification results from neighboring nodes (i.e., exploiting physical redundancy) to increase the classification reliability of individual sensors. Additionally, the whole system has been designed so it can meet the computing constraints typically found in the potential application domain of this system (i.e., low-cost WASN [15]). In order to assess the classification performance of the presented approach, real-world data have been collected simultaneously at four corners of a traffic intersection in Barcelona.

Overall, the contributions of this paper are the following:

- A new real-world 5 h length dataset (containing concurrent events) recorded simultaneously at four spots from a street intersection. This results in 4 × 5 h of acoustic data. A total of 5 h of audio data corresponding to 1 spot have been manually annotated. To the best of our knowledge, this is the first dataset with these characteristics.
- A software-assisted strategy to reduce the number of user interactions when labelling acoustic data to reduce the amount of time spent on this task.
- A two-stage acoustic classifier aimed at increasing the local classification robustness by taking into consideration the classification results of neighboring nodes (i.e., exploiting the nodes’ physical redundancy).

Conducted experiments over different low-cost architectures (Raspberry Pi 2B, 3B+, and 4) endorse the feasibility of our approach and encourage practitioners to extend this work in a large-scale real-world deployment.

The remainder of this paper is organized as follows. Section 2 reviews the related work on the identification of acoustic events in urban environments. Section 3 describes the real-world data collection and annotation processes that have led to the training and test sets used to assess the classification performance. Section 4 details the proposed two-stage multilabel classifier system. Section 5 evaluates the proposed approach. Section 6 discusses the main findings of this work. Finally, Section 7 concludes the paper and proposes potential future work directions.

## 2. Related Work

There is an increasing demand for automatic monitoring of noise levels in urban areas, especially if this monitoring can give information about the noise source of the measured levels [9,10]. In this sense, several WASN-based projects are being developed in several parts of the world, mainly adapted to their requirements, some of them identifying types of noise source and others giving equivalent levels $L_{Aeq}$. Following this idea, some projects have to develop their own sensors to meet the requirements of the measurements, and others operate in the real world with commercial sensors. Additionally, there are some projects that do not only concentrate on noise monitoring but also on air pollution.

### 2.1. Commercial Sensor Networks

Commercial sound level meters or sensor networks are usually connected to a central server, which collects all the $L_{Aeq}$ values gathered by the nodes. One of the first projects developed for this purpose is the Telos project [17], which was one of the first experiences in this WASN design by means of an ultra-low-power wireless sensor module designed by the University of California (Berkeley). Some years later, a WASN was used in a large variety of environmental monitoring applications, with a central focus on urban sound, as we can find in [18,19].

In Xiamen City (China), authors deployed a traffic noise monitoring network covering 35 roads and 9 green spaces in the city [20]. Data from the environmental monitoring stations were used to model the traffic of more than 100 roads in the city. Similarly, the FI-Sonic Project is focused on noise monitoring in a surveillance mode [21]. Its main goal is to develop the artificial intelligence algorithms required to identify the location of sound events [22] based on a FIWARE platform. The RUMEUR project, standing for Urban Network of Measurement of the sound Environment of Regional Use, is a hybrid wireless sensor network deployed by BruitPaif [23] in Paris and its surrounding cities. It has been designed to have high accuracy in critical places, such as airports, where the WHO directive has defined stringent thresholds [5], while other locations have less precise measurements. Years after, the RUMEUR project has evolved to Medusa [24], a new network combining four microphones and two optical systems with the goal of identifying the sound source location. Its computational load is high, and therefore it cannot be resolved by most of the low-cost acoustic sensor systems.

### 2.2. Ad Hoc Developed Acoustic Sensor Networks

Other projects have the goal of developing a custom WASN in order to meet the requirements of specific applications, mainly of particular analysis over the acoustic data. The IDEA project (Intelligent Distributed Environmental Assessment) [25] seeks to analyze air and noise pollutants in several urban areas of Belgium. It integrates a sensor network based on a cloud platform, and it measures noise and air quality [26]. The CENSE project, which stands for characterization of urban sound environments, is committed to conceiving noise maps in France [27], integrating both simulated and measured data collected from a cost-affordable WASN. The MESSAGE project, which stands for Mobile Environmental Sensing System Across Grid Environments, [28] not only monitors noise, carbon monoxide, nitrogen dioxide, and temperature, but also goes further and gathers real-time humidity and traffic occupancy in the United Kingdom. Moreover, the MONZA project [29,30] follows both the idea of monitoring urban noise real-time together with other air pollutants in the Italian city of Monza.

A more recent approach when working with WASN and noise sources is the hybrid approach of combining the acoustic information with subjective perception surveys that are specially focused on the typology of events affecting everyday life activities, such as sleeping or studying [31]. A noise identification system is applied to provide information about the detected sounds and establish a relationship between the perception surveys and the identified events related to road traffic noise [32].

One of the projects that faces the challenge of urban sound classification is Sounds of New York City Project (SONYC), which monitors the city using a low-cost static acoustic sensor network [33]. The goal of this project is to monitor noise pollution in real time by identifying the different noise sources that populate an acoustic environment. In this regard, it uses acoustic event detection [12,34] over all the collected (and annotated) urban acoustic data [35].

Another project with a similar conceptual background is the DYNAMAP project [36], which deployed two pilot areas in Italy, located in Rome [37] and Milan [38], with the idea of computing and comparing the noise impact of road infrastructures in suburban and urban areas, respectively. The two WASNs monitored road traffic noise by reliably collecting data at a frequency of 44,100 Hz, managing to remove specific non-traffic audio events [39,40] in order to build a more accurate road traffic noise map [41].

Deep learning has been applied to urban audio datasets, obtaining encouraging results [15,42]. However, many research studies are limited to datasets that are unrealistic because they are curated from audio libraries rather than real-world urban monitoring, and/or are single-label annotated, neglecting the simultaneous occurrence of sounds [43]. Recent work suggests that using multilabel data can enable practitioners to obtain more realistic results [42]. In this regard, edge intelligence is envisaged as a powerful alternative to address the typical computation overhead associated with multilabel classification systems [44].

### 2.3. Sensor Deployment Strategies

In addition to the sensors, an important design parameter for wireless (acoustic) sensor networks is the physical topology in which sensing units are deployed in a specific scenario. This section reviews the impact of the sensor deployment strategy on (1) the maximum size of the area of interest to be covered, (2) the power consumption of each node, and (3) the communication robustness.

As far as the area of interest is concerned, in [45], the authors study different node placements for a wireless sensor network able to sense environmental parameters (e.g., sunlight, temperature, humidity, rainfall, or images) that are delivered to different base stations by means of ad hoc wireless communication links. Concretely, the authors propose an analytical model to come up with the optimal position of nodes according to the desired node arrangement (e.g., ring, star, triangle, square, and hexagon). Alternative sensor location strategies have been studied for Underwater Acoustic Sensor Networks (UASN) applications as well. For instance, in [46], the authors study and compare the impacts of node deployment strategies in a 3-D environment. Their results show that a regular tetrahedron deployment scheme outperforms other topologies such as a random or cube topology. Concretely, the metrics that they use to compare the different schemes are the reduction of localization error and the optimization of localization ratio while maintaining the average number of neighbouring anchor nodes and network connectivity. Similarly, in [47], the effects of deploying UASNs together with the most well-known research projects in this field are reviewed.

Another way of extending the area size consists of using mobile nodes (e.g., robotic vehicles). For instance, in [48], the authors consider a dynamic topology in which nodes are constantly moving and study the best way to optimize power consumption.

Finally, in [49], the authors propose an advanced strategy for sensor placement that aims to maximize the connectivity robustness of the nodes for sparse networks. Concretely, they explore an analytical topology composed of hexagonal clusters and develop an algorithm for geometric distance optimization to improve the overall robustness of the system.

## 3. Collection and Annotation of a Real-World Dataset

To evaluate the results of a multilabel classifier, the first step is to have available a dataset with multilabel data. This section (1) describes the procedure that we followed to collect these data from a real-world environment, (2) details how data were labeled, and (3) exhibits the number of events for each class that were identified in the dataset.

### 3.1. Recording Campaign

In order to obtain a suitable real-world dataset to validate the proposed approach (i.e., multilabel classification of urban sounds taking advantage of physical redundancy in sensor nodes), two recording campaigns were conducted in the metropolitan area of the center of Barcelona (Spain). To have a wider variety of data, the two recording campaigns took place in different seasons of the year. The first one was conducted during autumn 2020 (17 November 2020) and the second one was conducted during spring 2021 (31 May 2021). Another substantial difference is that the first recording campaign was conducted under mobility restrictions [50] due to the COVID-19 pandemic, while during the second recording campaign, those restrictions were significantly softer. To have even more diversity in data, the hours in which the recording campaigns took place were different: whereas the autumn campaign was recorded from 12:00 to 14:30, the spring campaign was recorded from 15:30 to 18:00.

The location where the recording campaign was conducted is a specific crossroad in the Barcelona city center: the crossroad between Villarroel Street and Diputació Street (plus code 95M5+H9). This place is located in the Eixample area of Barcelona, which is the wide expansion district of the city. This place was chosen in order to validate the architecture proposed in [15], as its shape follows strictly regular symmetry. From now on, these recordings will be referred to as Eixample Dataset.

**Figure 1:**
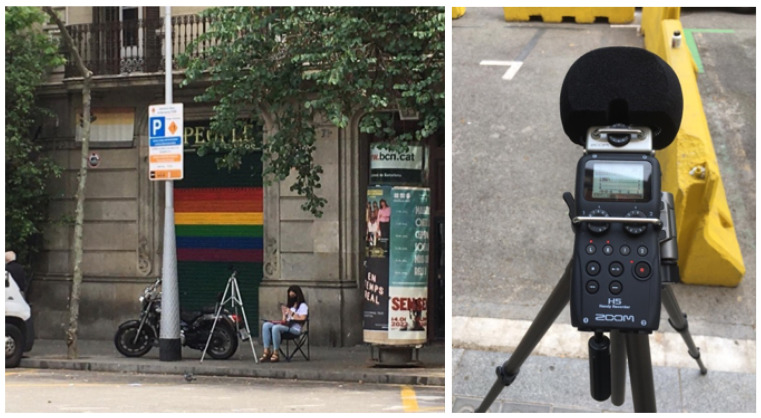
Recording campaign and Zoom recorder.

Four Zoom H5 recorders [51] (see Figure 1) were used to record data, with one placed on the middle of each corner of the street intersection. Concretely, the devices stood over tripods at a distance of at least 4 m from the closest wall and 1.5 m from the floor to avoid undesired sound reflections. Furthermore, the inclination of the device with respect to the floor was 45°. This will enable us to have simultaneous audio recordings in order to assess with real-world data whether physical redundancy helps increase the robustness of the classification results of the end-to-end architecture proposed in [15], as in that work we used synthetically generated audio files.

The two recording campaigns resulted in about 2 h and 30 min of acoustic data per sensor per campaign. Due to technical problems with the batteries of the recorders during the second campaign, the files were fragmented into two audio files. The time dedicated to changing the batteries was of about 5 min, in which we were not able to record data.

### 3.2. Data Labeling

Existing approaches to automatically label acoustic data [13] inspired by semi-supervised learning techniques shall not provide the necessary high level of accuracy and precision to train and test a reliable model to be considered as ground truth in order to assess our proposed approach. Therefore, data collected from the recording campaign have to undergo the manual labeling process described below.

Our previous experiences on (manually) labeling real-world acoustic datasets [52,53,54] taught us that assigning a tag to an acoustic sample is a time-consuming process: contrary to other types of datasets (e.g., images) in which a label can be assigned as soon as the sample is shown, in acoustic data labeling one has to wait for the whole acoustic record to be reproduced before assigning it a label. Typically, this is done with off-the-shelf software alternatives such as Audacity [55] that provide end-users with a spectrogram of the full audio record; thus, it becomes easier to visually identify those time frames in which something anomalous (i.e., potential events of interest) might be happening. However, as the purpose of this work is to identify multiple events (i.e., classes) that occur concurrently not only in the foreground but also in the background, and thus potentially overlap in a given acoustic sample, all of the collected acoustic samples—coming from the aforementioned two 2.5 h length campaigns—must be systematically heard and labeled.

In this situation, off-the-shelf software alternatives come at little ease due to the fact that they require, from the user point of view, several sequential and time-consuming interactions with the mouse (e.g., dragging and selecting the desired part of the spectrogram, clicking to add the label) and keyboard (e.g., typing the labels for the selected area). Additionally, as far as keyboard interactions are concerned, we have found that it is very common to make typos when writing the labels (e.g., typing *rnt* instead of *rtn*), which often require an additional review stage before feeding the labels to the machine learning system. Obviously, all these interactions add a significant delay on the overall data labeling process.

**Figure 2:**
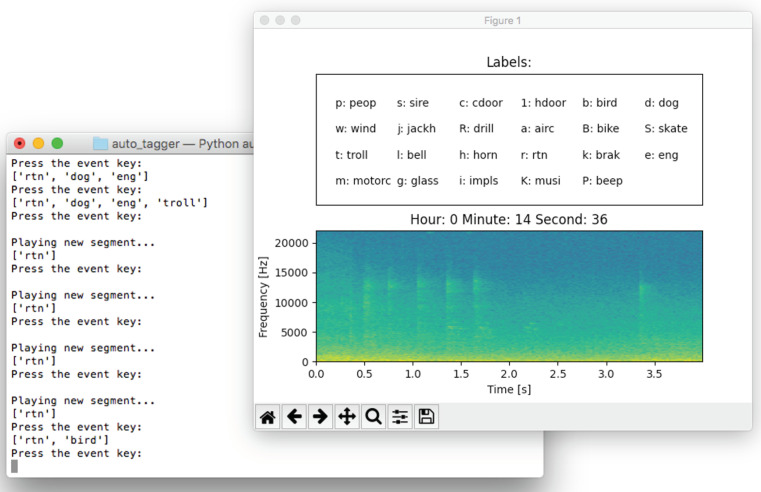
Screenshot of the developed python script. The screen on the background (**left**) records the keystrokes. The screen on the foreground (**right**) shows the information of the current window and a legend with the correspondences between keys and labels.

To address these shortcomings, we decided to develop a simple, yet custom, python script aimed to ease the manual acoustic data labeling process. The behavior of the script is described in the following points:

- Input. The script reads the .wav files coming raw from the Zoom H5 recorders. This done with the module AudioSegment of the pydub library. This module loads the whole audio is input into a vector, which results in a very convenient solution when windowing it.
- Configuration file. Moreover, the script reads a configuration JSON file specifying (1) the window size in which the .wav file will be split, (2) all the possible labels that may appear in the recording, and (3) a key (one letter long) associated with each possible label.
- User interaction. As shown in Figure 2, the script (1) displays a screen with the spectrogram—using the pyplot module of the matplotlib library—of the current window together with its start and finish times, (2) continuously reproduces the audio associated to the current window using the pyaudio library, and (3) shows the possible labels together with their associated keys in another screen. Then, each time the user presses a key corresponding to a label, the label is aggregated to the vector of labels associated with the current window. If the same key was pressed again, that event would be removed from the vector. Furthermore, the user can go to the following or previous acoustic window by using the arrow keys. Note that in this way, the user has a single interaction device (i.e., keyboard) and typos in labels are not possible.
- Output. The script writes a .csv file with (1) the start time of the window, (2) the finish time of the window, and (3) all the tags that have been selected for that window. For instance, a line in this .csv file would appear as follows:
  276.000000 280.000000 bike+dog+troll 280.000000 284.000000 bike+glass  284.000000 288.000000 dog+peop+drill
  As a result, each line of the labels file derived from a recording contains the starting and ending time of the window and the different labels assigned to (i.e., appearing) that fragment.

Thanks to this software, we experienced that roughly, on average, the labeling process took us 30% less time than what it took in other works where we used off-the-shelf alternatives.

As far as the data labeling process is concerned, we labeled the acoustic data (i.e., two 2.5 h length recordings) from one of the four corners of the recording campaign. Concretely, it took about 12 h to annotate all these acoustic data using the aforementioned method. These data were then used as a reliable ground truth for the experimental evaluation. The other audio files were not manually labeled as they were only used as a complement to the selected sensor to check if the accuracy improves when joining together the classification results from neighboring nodes. In order to use a classification algorithm based on a deep neural network able to classify the spectral information of acoustic data [15], we decided to directly label the audio files in windows of 4 s to keep compatibility with previous experiments [15]. Hence, as it can be seen in the spectrogram depicted in Figure 2, the script sequentially split audio files in windows of 4-s length.

### 3.3. Obtained Dataset

The manual labeling task led the team to this taxonomy, with the number of classes and the number of labeled events shown in Table 1.

### 3.4. Train/Validation/Test Split

As can be seen in the last column of Table 1, the dataset is highly imbalanced: whereas the top events of the table are present in both recording campaigns with a considerable number of samples, there are some sounds that are poorly represented in the dataset. Actually, there are acoustic events that are only present in one of the two recording campaigns. For example, the *drill* sound is present only in the second campaign. Moreover, as the events were labeled in 4-s windows, the fact that there are 14 events labeled with the *drill* tag does not mean that there are 14 independent drilling sounds, as a long drilling sound lasting (for example) 8 s would be counted as two different windows. This phenomenon would happen with all the acoustic events that last more than the 4-s window (presumably, categories such as *sire*, *musi*, *eng*, or *motorc* among others). We are took this into account when splitting the dataset in Train/Validation and Test sets (see Section 3.4).

## 4. Two-Stage Multilabel Classifier

After obtaining the labeled dataset, this section details the whole classification procedure. First, it discusses the feature extraction process of the acoustic data. Next, it shows how the dataset is split into Train, Validation, and Test sets. Then, it describes how the problem of class imbalance has been addressed by using data augmentation. Finally, it details the two-stage classification process.

### 4.1. Feature Extraction

As features, and to maintain compatibility with [15], a spectrogram was obtained from each 4-s window of the dataset. Audio files were originally recorded at a sampling rate of 44,100 Hz. First, we considered down-sampling the audio files to 22,050 Hz, but after analyzing the labeled events, we realized that the *brak* event had all its frequential information at the band of ∼17,000 Hz. Considering the Nyquist theorem, if the *brak* event is aimed to be detected, a sampling rate of 22,050 Hz is not high enough. Hence, we finally decided to keep the original 44,100 Hz frequency, even if it required more computational resources.

Each spectrogram was generated with a Fast Fourier Transform (FFT) [56] window of 1024 points and using the librosa python library [57]. Next, each spectrogram was individually normalized to have a minimum value of 0 and a maximum value of 1 for compatibility with the input format of the neural network.

The audio files obtained on the recording campaign had to be divided into Train, Validation, and Test subsets. As soundscapes have temporal continuity, and so to evaluate the machine learning algorithm correctly, it is important to make sure these three data subsets are taken from different moments of the day, so that one single event is not split into different groups. Therefore, we tried to avoid or mitigate the fact that different audio samples with similar background noise were placed, for example, on both the Training and Testing sets.

**Figure 3:**
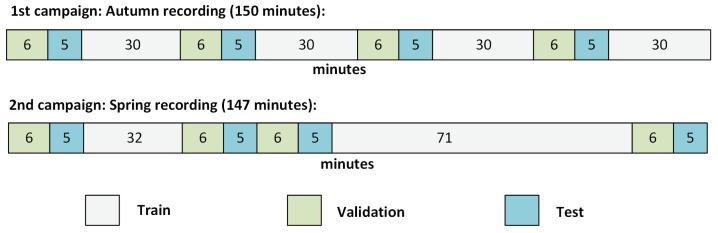
Duration and temporal splitting of the Train, Validation, and Test sets of the dataset.

Concretely, the division was done as shown in Figure 3: with divisions into contiguous regions ranging from 5 to 71 min length.

This division left the dataset with 209 min for Training, 40 min for Validating, and 48 min for Testing. Note that the division of the two datasets was not exactly even due to the distribution of the events. We tried to maximize the variety of the events on each of the datasets while keeping their temporal evolution.

As can be appreciated in Table 2, the three sets are highly unbalanced. Note that due to the lack of drilling events during the recording campaigns (only 14 consecutive events), we were unable to test that category properly. We discarded the option of splitting the 14 events into the Train and Test sets as they belonged to the same drilling machine recorded in the same location, which may have generated biased results. Moreover, we decided to remove the *cmplx* sounds from the dataset. As we could not identify the specific source of those sounds when labeling them, we arrived at the conclusion that they may confuse the system.

### 4.2. Data Augmentation

To mitigate the potential effects of class imbalance while training, we decided to add more training data and to apply data augmentation techniques to obtain more samples on the poorer classes. Additional data were obtained from the BCNDataset [52], which is a dataset containing real-word urban and leisure events recorded at night in Barcelona. As the BCNDataset was labeled differently than the Eixample Dataset, labels from both datasets were unified.

More concretely, per each of the acoustic events, on the BCNDataset the labels are provided as:

start_second end_second label

Note that in BCNDataset, the difference between the starting time and the ending time of each acoustic label is variable (not as in Eixample Dataset, where the ending time is always 4 s later than the starting time), and only one label is provided per each row of the text file. However, the format of the file is the same as the one presented in Section 3.2, which eased the merging process of both datasets. To merge both datasets, the labels of the BCNDataset were fragmented and grouped in windows of 4 s. This way, we were able to obtain one-hot encoded multilabel labels.

**Figure 4:**
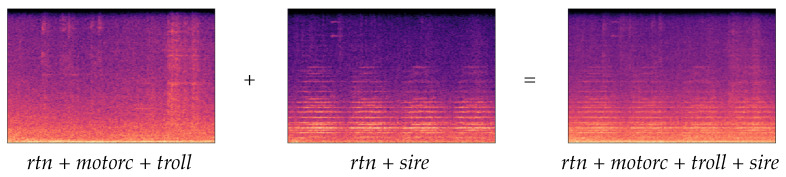
Example of mixup data augmentation using two random 4-s fragments containing several acoustic events.

The concrete data augmentation technique used in this work consisted of audio mixing, sometimes known as mixup [58]. As shown in Figure 4, two spectrograms (one belonging to the Eixample Dataset and the other belonging to BCNDataset) were added and then divided by two to maintain 0-to-1 normalization values. As the newly generated sample would contain information of all the events tagged in both spectrograms, the labels file was generated by aggregating the one-hot encoding values as well. This process was carried out using pseudo-random spectrogram selection until all the classes had about 500 samples on the Training set.

### 4.3. Multilabel Classification

The classification process consists of two layers:

1. The first layer (Section 4.3.1) is a Deep Neural Network (DNN) that classifies 4-s fragments in a single node.
2. The second layer (Section 4.3.2) aggregates the classification results of the deep neural networks running on the four corners of the intersection and makes a final decision on what events are actually happening on each corner by means of an ensemble of classifiers.

#### 4.3.1. First Stage: Classification in One Node

**Figure 5:**
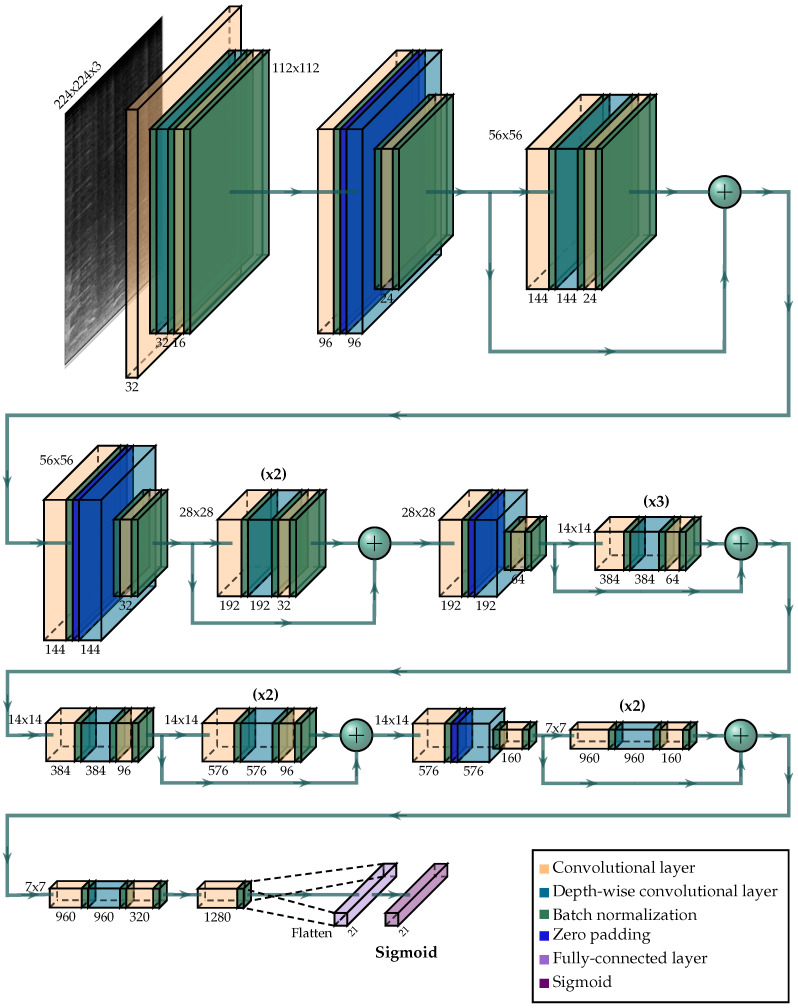
Architecture of the MobileNet v2 [59] deep neural network used at the first stage of the classification process.

The classification of the events on each of the nodes was carried out using a deep neural network with a MobileNet v2 architecture [59] with a size of 8.8 MB—which should fit on a low-cost computing node for a WASN. As shown in Figure 5, the last layer of the neural network was replaced by a fully connected layer with one neuron per class and a Sigmoid activation function on each of them to allow multilabel classification. As a result, for each input datum, the output neurons showed the probability of that class being present on the input spectrogram. Once the probabilities were obtained, to evaluate whether the deep neural network was able to classify correctly without taking into account the decisions made by neighboring nodes, custom thresholds for each class were applied to determine if the event was actually present on the 4-s fragment. The thresholds were obtained by maximizing the F1-measure of each class on the validation set. As hyperparameters, an ADAM optimizer [60] was used with a learning rate of 1 × 10${}^{-4}$ and a weight decay regularization of 1 × 10${}^{-5}$.

#### 4.3.2. Second Stage: Classification Using Physical Redundancy

The aim of the second classification stage is to increase the robustness of the classification conducted at the previous stage by exploiting the physical redundancy of the nodes (i.e., nodes are physically deployed in such a way that the same event can be listened to by more than one node). Robustness in this context refers to the ability of the classifier to perform correctly when the output probabilities of the deep neural network for a given class are low but the event is actually happening. In this regard, our proposed system takes into consideration the classification results of neighboring nodes in order to strengthen (or weaken) its own results. For instance, if in the same frame Node A classified a *bell* with probability 0.3 and nodes B, C, and D classified *bell* with probability 0.8, then Node A should infer that a *bell* event actually happened. As manually defining these thresholds (or rules) might disregard some of the internal dynamics of the system, we propose to use a classifier to automatically generate them.

The process followed to train the automatic second stage classifier is detailed in what follows:

1. Once the deep neural network was trained, we used it to obtain a 21-component classification vector per each of the 4-s fragments of the original Eixample Dataset (see Section 3). Each component of the vector indicated the likelihood of an acoustic event being present on the fragment. The labels from the dataset associated with each fragment were kept as ground truth.
2. The previous stage was done with the simultaneous audio of the remaining three neighboring locations. Therefore, for each 4-s fragment of the Eixample Dataset, we obtained four 21-component vectors together with the ground-truth labels.
3. The four vectors were concatenated horizontally, thus obtaining a single 84-component vector.
4. The 84-component vector and ground truth labels were used to fit a machine learning model that would output the final classification results.

**Figure 6:**
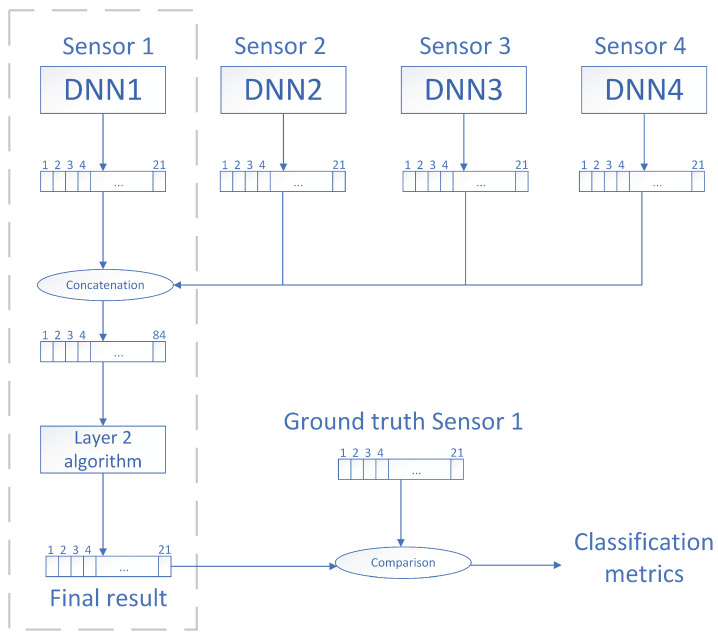
Proposed system architecture with two classification stages. The first deep neural network of the first level outputs a 21-component vector that is later concatenated with the vectors from neighboring nodes. The resulting 84-component vector is examined by the second classification stage to obtain the final classification result. This scheme is replicated on each of the sensors of the system.

For more clarification, this procedure is illustrated in Figure 6.

## 5. Experimental Evaluation

To assess the classification performance of the proposed system, each one of both classification stages was evaluated.

### 5.1. Classification Performance at the First Stage

We evaluated the effect of training data on classification performance of the deep neural network. Concretely, four experiments were conducted, differing only in the datasets used for training:

- **Experiment 0**: We used the Training set of the Eixample Dataset and the entire BCNDataset without using data augmentation techniques.
- **Experiment 1**: We used the Training set of the Eixample Dataset and the entire BCNDataset using the data augmentation techniques detailed in Section 4.2 to have around 500 samples for each class.
- **Experiment 2**: We used the same data as in Experiment 1 and we also added data from the UrbanSound 8K dataset [43]. The sampling frequency of most of the audio files of the UrbanSound dataset is lower than the one used on the recording campaign (i.e., 44,100 Hz). In order to avoid having half of the spectrogram empty for the UrbanSound samples, each audio file was combined with an audio file from Experiment 1 using mix-up aggregation (that is, two spectrograms are aggregated, each of them having a different weight on the final image). Concretely, the audio files from the UrbanSound 8K dataset were only assigned between a random 10% to 30% on the final weight of the spectrogram.
- **Experiment 3**: We used the same data as in Experiment 2, but on this occasion, each audio file from the UrbanSound dataset was used 10 times to combine it with a different audio file randomly selected from the BCNDataset or the Eixample dataset. This way, we increased the size of the Training data.

The metrics that we used to compare the results are the Macro and Micro average F1-scores [61]. Whereas the first metric gives an overall classification result without taking into account the number of samples of each class (i.e., all the classes have the same importance), the second one considers the number of samples of each class of the dataset (i.e., those classes that have a greater number of samples on the Test set have more importance). We present both results because, on the one hand, the macro average could be biased because of the limitations of the Test set in some classes (e.g., there is only one dog event, which means that the F1-measure for that class will be binary); on the other hand, the micro average could be biased as well as the *rtn* class is present in almost all the audio samples. Hence, whereas the first metric is mostly affected by the performance of the smaller classes of the dataset, the second one is mostly affected by the performance of the larger classes of the dataset. Table 3 shows the classification results for each of the experiments. To compute the classification metrics, the *drilling* class was not taken into consideration as there are no events from that class on the Test set.

As can be seen in Table 3, using the imbalanced data from the BCNDataset and the Eixample dataset without using any data augmentation techniques (i.e., Experiment 0) results in poor classification results. Concretely, the 12% on the Macro average F1 score tells us that the algorithm has problems in classifying most of the categories. In addition, having a Micro average F1 score higher than the Macro average F1 score tells us that the system performs better when classifying those categories with more samples than when classifying those categories with few instances. This phenomenon can be appreciated in all the experiments.

Generally speaking, as shown in Table 3, the data augmentation techniques that have been used in this work (i.e., Experiments 1, 2, and 3) have helped build a more robust system. However, we think that using the UrbanSound samples has not actually helped to improve the performance of the overall system at all due to the following reasons. First, even if UrbanSound is a balanced dataset, it has fewer categories than the Eixample dataset. In addition, the difference between the original sampling rate of the Eixample dataset or the BCNDataset and the audio files from the UrbanSound dataset resulted in less realistic audio files than if we used two real-world datasets recorded with similar conditions (as we did in Experiment 1). Actually, comparing the classification metrics from Experiment 2 and Experiment 3, we can see that Experiment 2 has a better performance. We think that this is because when doing data augmentation, only a random 10% to 30% of data belong to the UrbanSound dataset, which means that most of the information belongs to the spectrograms from the other two datasets. As we are augmenting data 10 times using the same base spectrograms, the deterioration of the classification results may indicate that we are biasing the deep neural network with mild overfitting towards these base spectrograms when training.

To sum up, we propose that the data used in Experiment 1 offer the fairest trade-off between the performance of the system on large and small classes. Hence, from now on, for the experiments performed on Stage 2, we will use the model trained with Experiment 1 data.

### 5.2. Classification Performance at the Second Stage

As in this work the only labeled data that we had available were the ones recorded on one specific sensor, the experiments were conducted over that reference sensor. To discover the most suitable machine learning algorithm for the second classification stage, four different classification algorithms were evaluated:

1. Decision Tree (DT): The size of the model after training was 617 KB.
2. Random Forest (RF): The size of the model after training was 121 MB.
3. Logistic Regressor (LR): The size of the model after training was 20 KB.
4. XGBoost (XGB): The size of the model after training was 2.3 MB.

It is worth noting that the lighter classification algorithms from a computing point of view (i.e., the ones that require less RAM) are the DT and the LR, followed by XGB and, finally, the RF. In this case, to build the models, the only data that we could use were the ones belonging to the Eixample dataset, as this is the only one that has four simultaneous recordings. The algorithms resulted in the classification results shown in Table 4. As classification metrics, apart from the metrics shown in Section 4.3.1 (i.e., Micro F1 average and Macro F1 average), the Micro precision and Micro recall of the system are shown as well [61].

As can be seen in Table 4, all the algorithms tend to have slightly higher values of Micro precision than Micro recall, which are emphasized in some of the classifiers (i.e., RF and XGB). Whereas the first metric illustrates what proportion of detected events were actually correct, the second one shows what proportion of actual events were correctly classified. The Macro F1 measure gives the same importance to both metrics.

Whereas the highest Micro precision result is achieved by using the RF algorithm (81.8%), the highest Micro recall result is obtained using the LR (72%). However, we believe that for the current context of this work (i.e., classification of urban sounds), we should also consider the F1 scores. In this sense, for the Micro F1 score, three classification algorithms present similar results (the RF, LR, and XGB with 74.3%, 74.6%, and 74.1%, respectively). However, when checking the Macro F1 average, XGB outperforms the other classification algorithms, obtaining a final score of 39.3%. Therefore, we believe that the algorithm that presents the fairest trade-off between all the classification metrics is XGB.

When comparing the classification results obtained at the first stage to the classification results obtained at the second stage, we can see that using physical redundancy allowed for increasing the F1 Macro average from 70% to 74.1% (+4.1%). Regarding the F1 Micro average, the results change from 39% to 39.3% (+0.3%). These increments suggest that the second stage helps to improve classification results mainly on the classes that have more instances.

As this system is to be deployed in a low-cost device such as the one presented in [15], it is not only the accuracy that matters but also the capability of the system in making real-time classifications within the 4-s selected window. Moreover, to make the classification process smoother, it would be desirable to use a sliding 4-s window with hops as small as possible (i.e., obtaining as many classification results as possible by sliding the 4-s window with overlap). The amount of overlap that can be used in the system depends on the classification speed of the system to output new data.

For this reason, to check the amount of time that it would take to the system to output a new classification result, experimental tests were carried out using three different computation units (i.e., Raspberry Pi Model 2B, Raspberry Pi Model 3B+, and Raspberry Pi Model 4) and a plug-and-play USB microphone.

The main hardware differences among these three models relevant to the research presented in this work are their computation capabilities (central processing unit and operating frequency) and their amount of RAM memory:

- Raspberry Pi Model 2B: Broadcom BCM2836 SoC (ARMv7), Quad-core ARM Cortex-A7, @ 900 MHz, 1GB LPDDR2 of RAM.
- Raspberry Pi model 3B+: Broadcom BCM2837B0 SoC (ARMv8), Cortex-A53, 64-bit @ 1.4GHz, 1GB LPDDR2 SDRAM.
- Raspberry Pi model 4: Broadcom BCM2711 SoC (ARMv8), Quad-core Cortex-A72 64-bit @ 1.5GHz, 4GB LPDDR4-3200 SDRAM.

For each experiment, we evaluated the timing performance of the processing units by making 100 test runs on each device. The obtained results can be seen in Table 5. The times on the table start counting since a 4-s fragment is acquired by the microphone, and they include (1) the spectrogram computation, (2) the first stage classification (DNN), and (3) the second stage classification. As can be observed in the table, the device in which the experiments are conducted greatly affects the timing results.

Even though all the Raspberry Pi models are able to obtain a classification result within 4 s and would hence be suitable for a real-world deployment of the system, Raspberry Pi Model 2B offers a timing response that is at least about 1 s slower than its superior models. It can also be observed that Raspberry Pi Model 4B is, in general, about 0.5 s faster than Raspberry Pi Model 3B+. Concretely, when using Raspberry Pi Model 4B, the average response time of the system to perform a complete classification ranges from 0.66 s (when using the DNN + DT) to 0.78 s (when using the DNN + RF). Concretely, when using the aforementioned DNN + XGB algorithm, the classification would take on average 0.77 s. In this case, the system could use a 4-s length sliding window and a hop of 1 s (i.e., maximum classification time for DNN + XGB in Model 4B) and thus output a classification result in the next second.

Finally, to observe with detail the classification results obtained when using the selected parameters, Table 6 shows the individual classification metrics per each class of the dataset based on the results obtained in Experiment 1 on Section 4.3.1 and using the XGBoost classifier. As can be seen, the system has a good performance when classifying events with more than 100 instances on the Validation and Test set (values highlighted in Table 6). However, it behaves poorly when classifying those classes with few instances except for the bell event. This may be due to the fact that in the recording location, the saliency of the recorded bells was higher than the background noise, so all the recorded bells are foreground events. On the contrary, events such as sirens or music were occasionally mixed with background noise depending on the distance between the noise source, the sensor, and the simultaneous acoustic events happening at the same time.

## 6. Discussion

From our point of view, we believe that the obtained results in this research are encouraging in terms of covering the expected results. In fact, the proposed system has been shown to properly operate in a real-world environment. That is, the proposed system has been exposed to the real-operation conditions (in terms of audio) typically found in urban environments: appearance of sounds not previously recorded, various events happening simultaneously, etc. Using inexpensive commodity hardware (i.e., less than EUR 100 Raspberry Pi Model 4B), it has been able to produce classification outputs with reasonable accuracy in 1 s.

### 6.1. Location Perspective

The intrinsic Eixample topology makes the deployment of the sensors straightforward for this specific scenario. As all streets are totally symmetrical in this part of Barcelona, it is possible to deploy one sensor in each corner of the crossroads. If the proposed system were extended to the whole city, the symmetry for this low-cost sensor network would be still guaranteed for all places in the city center. However, up to now, we thought we should analyze, or at least test, the results with other distributions, also taking advantage of the symmetry of the streets. As we have found that the most relevant sounds are detected in most of the four sensors in a crossroad, it might be interesting to discover what would happen if the location is slightly farther or if the sensor deployment strategy is different. In the latter case, we envisage a design trade-off between the advantages of physical redundancy in terms of accuracy, the cost of the WASN (i.e., number of sensors), power consumption, robustness, and size of the area under interest.

**Figure 7:**
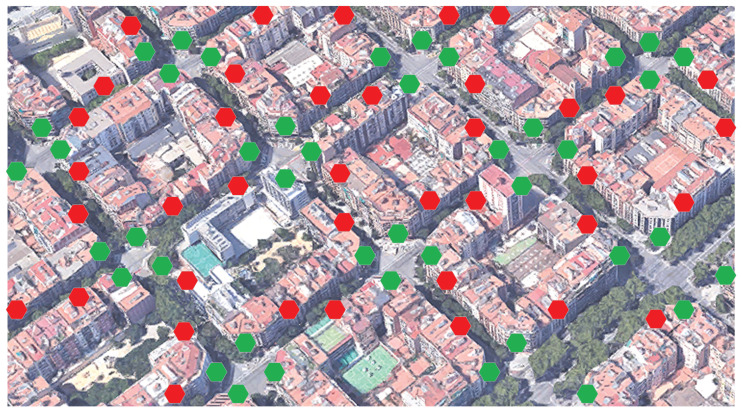
Example of a possible future location of sensors. Green dots indicate the location used for the experiments conducted in this paper. Red dots indicate the new proposed locations.

In Figure 7, we show a possible future location deployment of the sensors with a wider distance between them, which despite reducing the effects of physical redundancy, may make the nodes more aware of what happens in the streets, instead of focusing on the crossroads. This could open further research on discovering the optimal distance between sensors according to the symmetry of the streets and balancing accuracy with the number of sensors to be deployed. A further step in this analysis would be to study the deployment of the proposed system in other parts of Barcelona without the Eixample symmetry: narrower streets, irregular crossroads, small squares, and other urban layouts that may make distribution of sensors difficult, in order to cover all the events happening on the street.

An accurate identification of sounds in an urban acoustic soundscape taking advantage of physical redundancy in the nodes could help to locate the sound source. While this might not be relevant in some cases/applications, it could be really helpful when assessing noise complaints (neighbors, dogs, etc.). In fact, this could help local authorities to identify those places (buildings, shops, bars, discos, etc.) where the noise is generated and conduct appropriate corrective measures. Additionally, this could provide crucial support to model the noise behavior in any city if the proposed system is deployed for long periods of time (i.e., months or years). In this case, it would be possible to discover recurrent patterns as certain types of noise would come always from the same places (e.g. ambulances, trucks, motorbikes, etc). Hence, a city noise model could be designed using the outputs of the proposed system.

### 6.2. Accuracy and Sample Availability

According to the conducted experiments, we have observed that the F1-Micro average score is consistently higher than the F1-Macro average. This means that the system has better performance on those classes that have a significant number of instances for train and test. For instance, while the bird class has 913 instances for train and 208 instances for test, obtaining an F1-score of 0.78, the bike class only has 55 instances for train and 12 instances for test, obtaining an F1-score of 0. To fight this situation, we believe that the individual detection may be improved by balancing and obtaining more data from recording campaigns in the same location or in other locations in Barcelona.

However, the number of instances is not the only relevant factor here: as it can be seen in Table 6, the peop class has 954 instances for train and 181 instances for test, but only obtains an F1-score of 0.55. This drives us to think that the saliency of each event should be considered as well. In fact, we have observed that those events with low saliency are easily masked by other events occurring concurrently with higher saliency. This situation makes the system obtain a higher number of false negatives than false positives for those specific events. Further experimentation with alternative features and/or distinguishing between foreground and background events at the annotation stage would be needed to validate this hypothesis.

## 7. Conclusions

In this work, progress has been made in the Training, Testing, and Validation of a two-stage classifier composed of a deep neural network and an XGBoost classifier with a very relevant focus on the use of real-world data. In our experiment, real-world data gathered at the city center of Barcelona have been used to validate the feasibility of a real-operation deployment of the algorithm. The data gathering process has been carried out in four simultaneous spots at a traffic intersection in order to assess up to what extent physical redundancy increases the robustness of the classifier. Furthermore, a new data labeling procedure aimed to reduce the amount of time spent on the task of manually labeling acoustic samples has been described. We have also shown which strategies we used to enrich the gathered data (i.e., data augmentation) to balance the corpus and thus improve the performance of the classifier.

From the experiments conducted, we can conclude that applying data augmentation techniques has helped the classifier to identify better those categories with few instances on the dataset. Moreover, physical redundancy of sensors has helped increasing the Micro and Macro F1-metrics. However, the improvement is mostly noticeable in those classes of the dataset that have more sample instances.

A real-world deployment of a WASN capable of detecting multiple acoustic events occurring simultaneously such as the one proposed in this paper would enable public administrations to have more information available about the types of sounds present in each area of the city in real time. This information may be helpful to assess neighbor complaints or detect the most acoustically polluted areas as well as to design policies to improve the quality of life of citizens of the more acoustically polluted areas.

As future work, we foresee that adding a memory layer to the system may increase the classifier performance (e.g. if there is a *siren* sound in a 4-s fragment, then it is likely that the next 4-s frame contains a *siren* sound as well). That is, we believe that knowing the probability of certain events in certain cases may help. Thus, this hypothesis will be further evaluated in future works. In addition, as it has been detected that the class imbalance of the dataset deteriorates the performance of the system on the poor classes, new training and testing data should be acquired. Finally, as the type of acoustic events present in urban environments are volatile, may vary day by day, and in some cases, only a few instances of each class might occur, it would be interesting to study the potential application of techniques that explicitly allow for new categories, such as few-shot learning or active learning.

## Figures and Tables

**Table 1:** Number of events annotated on the dataset.

		Number of Occurrences		
Label	Description	1st Campaign	2nd Campaign	Total
*rtn*	Background traffic noise	2177	2118	4295
*peop*	Sounds or noises produced by people	300	612	912
*brak*	Car brakes	489	424	913
*bird*	Bird vocalizations	357	960	1317
*motorc*	Motorcycles	769	565	1334
*eng*	Engine idling	203	913	1116
*cdoor*	Car door	133	161	294
*impls*	Undefined impulsional noises	445	170	615
*cmplx*	Complex noises that the labeler could not identify	85	73	158
*troll*	Trolley	162	152	314
*wind*	Wind	8	23	31
*horn*	Car or motorbike horn	43	33	76
*sire*	Sirens from ambulances, the police, etc.	18	57	75
*musi*	Music	8	30	38
*bike*	Non-motorized bikes	51	24	75
*hdoor*	House door	25	60	85
*bell*	Bells from a church	24	27	51
*glass*	People throwing glass in the recycling bin	17	32	49
*beep*	Beeps from trucks during reversing	31	0	31
*dog*	Dogs barking	3	25	28
*drill*	Drilling	0	14	14

**Table 2:** Number of events on the Train, Validation, and Test set.

	Dataset		
Label	Train	Validation	Test
*rtn*	3029	583	683
*peop*	954	100	181
*brak*	627	137	149
*bird*	913	196	208
*motorc*	954	183	197
*eng*	864	73	179
*cdoor*	190	51	53
*impls*	457	67	91
*cmplx*	128	16	14
*troll*	229	53	32
*wind*	19	4	8
*horn*	49	17	10
*sire*	69	0	6
*musi*	34	0	4
*bike*	55	8	12
*hdoor*	65	12	8
*bell*	34	4	13
*glass*	40	6	3
*beep*	9	13	9
*dog*	23	4	1
*drill*	14	0	0

**Table 3:** Macro and micro average F-1 scores for the experimental evaluation obtained at the first classification stage.

Dataset Used	F1-Macro Average	F1-Micro Average
Experiment 0	12%	46%
Experiment 1	39%	70%
Experiment 2	36%	75%
Experiment 3	33%	67%

**Table 4:** Experiment results obtained at the second classification stage.

Algorithm Used	Micro Precision	Micro Recall	Micro F1	Macro F1
DT	71.6%	69.5%	70.5%	30.6 %
RF	81.8%	68.1%	74.3%	26.7 %
LR	77.3%	72%	74.6 %	37.8%
XGB	78.5%	70.2 %	74.1 %	39.3 %

**Table 5:** Time that it takes for the system to classify a 4-s audio fragment using three different sensor models. Results are shown in seconds after 100 runs.

Algorithms	RPi Model	Max. Time	Min. Time	Avg. Time
		(seconds)	(seconds)	(seconds)
DNN + DT	Model 2B	2.3	2.0	2.2
DNN + RF		2.9	2.4	2.6
DNN + LR		2.4	2.0	2.2
DNN + XGB		2.8	2.4	2.5
DNN + DT	Model 3B+	1.3	0.9	1.1
DNN + RF		1.5	1.2	1.3
DNN + LR		1.3	1.1	1.2
DNN + XGB		1.4	1.3	1.5
DNN + DT	Model 4B	0.7	0.6	0.6
DNN + RF		0.8	0.7	0.7
DNN + LR		0.8	0.6	0.6
DNN + XGB		1.0	0.7	0.7

**Table 6:** Evaluation metrics of the system when combining the outputs of 4 local nodes by using the XGBoost algorithm.

Label	True Negative	False Positive	False Negative	True Positive	F1-Score
*rtn*	0	37	11	672	0.97
*peop*	495	44	96	85	0.55
*brak*	513	58	80	69	0.50
*bird*	485	27	59	149	0.78
*motorc*	469	54	79	100	0.60
*eng*	502	39	41	138	0.78
*cdoor*	652	15	40	13	0.32
*impls*	598	31	61	30	0.39
*troll*	670	18	18	14	0.44
*wind*	709	3	5	3	0.43
*horn*	709	1	7	3	0.43
*sire*	701	13	5	1	0.10
*musi*	714	2	4	0	0
*bike*	707	1	12	0	0
*hdoor*	705	7	8	0	0
*bell*	707	0	6	7	0.70
*glass*	707	0	2	1	0.50
*beep*	711	0	9	0	0
*dog*	718	1	1	0	0

## Abbreviations

The following abbreviations are used in this manuscript:

DNN	Deep Neural Network
DT	Decision Tree
EEA	European Environment Agency
EU	European Union
LR	Logistic Regressor
RF	Random Forest
UASN	Underwater Acoustic Sensor Networks
WASN	Wireless Acoustic Sensor Network
XGB	XGBoost
WHO	World Health Organization
